# Comparative outcomes of delta-on-delta and delta-on-highly cross-linked polyethylene bearings in total hip arthroplasty: a nationwide cohort study

**DOI:** 10.1007/s00402-026-06502-1

**Published:** 2026-09-18

**Authors:** Young-Kyun Lee, Danbee Kang, Hyunsoo Kim, Eunjee Kang, Juhee Cho, Jung-Wee Park

**Affiliations:** 1 Department of Orthopaedic Surgery, Seoul National University College of Medicine, Seoul, Korea, Republic of; 2 Department of Orthopaedic Surgery, Seoul National University Bundang Hospital, Seongnam, Korea, Republic of; 3 Department of Clinical Research Design and Evaluation, SAIHST, Sungkyunkwan University, Seoul, Korea, Republic of; 4 Center for Clinical Epidemiology, Samsung Medical Center, Seoul, Korea, Republic of

**Keywords:** Total hip arthroplasty, Ceramic-on-ceramic, Ceramic-on-polyethylene, Complication, Survivorship

## Abstract

**Introduction:**

The purpose of this study was (1) to evaluate the national trends of Delta ceramic and highly crosslinked polyethylene (HXLPE) liner in total hip arthroplasty (THA), and (2) to compare survivorship of Delta ceramic versus HXLPE liner when used with a Delta ceramic head.

**Materials and methods:**

Using the K-NHIS database, we identified all patients who underwent primary THA using Delta ceramic head in South Korea between 2003 and 2013 (*n* = 16,250). Cumulative incidences of dislocation, periprosthetic joint infection (PJI), and periprosthetic fracture were evaluated according to bearing type: Delta-on-Delta ceramic (DoD) and Delta-on-HXLPE (DoHXLPE). Survivorship for revisions was calculated to determine differences. Multivariable Cox proportional hazard regression models were used for comparisons, and propensity score matching was additionally performed as a sensitivity analysis.

**Results:**

A total of 15,299 patients underwent THA with Delta ceramic head between 2003 and 2013. Delta liner was used in 14,255 patients (93.2%) and HXLPE in 1,044 patients (6.8%). Utilization of Delta liner has noticeably increased since 2007. The DoHXLPE group had a higher risk of PJI (aHR = 2.10, 95% CI = 1.29–3.41) compared to the DoD group within 1 year. There were no significant differences in risk of dislocation (aHR = 1.41, 95% CI = 0.97–2.05) and periprosthetic fracture (aHR = 0.90; 95% CI = 0.39, 2.07) within 1 year. After 10 years, the DoHXLPE group had a higher risk of any revision (aHR = 1.32; 95% CI = 1.03, 1.38) compared to the DoD group, despite the similar risk of cup revision and stem revision. In the propensity score–matched cohort, the increased risk of PJI remained significant, whereas the point estimate for any revision remained higher in the DoHXLPE group but was no longer statistically significant.

**Conclusion:**

DoHXLPE bearings were associated with a higher risk of PJI than DoD bearings. However, the difference in the risk of any revision was not statistically significant in the propensity score–matched analysis.

**Supplementary Information:**

The online version contains supplementary material available at https://doi.org/10.1007/s00402-026-06502-1.

## Introduction

Total hip arthroplasty (THA) is a promising procedure with long-term survival rates of more than 90% at twenty years of follow-up [1, 2]. Despite the effectiveness of this procedure, some patients often require revision surgery after THA [3, 4]. Currently, aseptic loosening of components is the most frequent cause of revision after THA [3, 5]. Osteolysis and subsequent aseptic loosening of components can be generated by polyethylene (PE) particles as a result of UHMWPE wear [5–7]. To reduce wear and wear-related osteolysis, ceramic and highly cross-linked polyethylene (HXLPE) bearings have been introduced as alternatives to conventional polyethylene [8–10]. 

Ceramic bearings have theoretical advantages, including high hardness, a smooth surface finish, and favorable wettability, which may reduce friction, wear, and osteolysis compared with conventional PE [9]. Although ceramic fracture was a concern with earlier-generation ceramics [9], fourth-generation BIOLOX Delta ceramic was developed to improve fracture resistance, and favorable outcomes have been reported [11–13]. Nevertheless, liner malseating remains a risk factor of ceramic liner fracture [14, 15]. 

HXLPE, introduced as another alternative bearing in THA, has been established by exposing UHMWPE to gamma radiation and/or thermal treatments to reduce wear and debris-induced osteolysis following THA [8, 10, 16]. 

Some studies using Western registry database have compared ceramic-on-ceramic (CoC) and ceramic-on-HXLPE (CoHXLPE) THA [17–19]. However, there has been lack of study to compare directly safety and effectiveness between Delta ceramic liner and HXLPE liner in THA with Delta ceramic head in a large East Asian nationwide cohort.

The purposes of the study were (1) to evaluate the current trend of utilization of Delta-on-Delta (DoD) and Delta-on-HXLPE (DoHXLPE), and (2) to compare the survivorship of each type of bearing, using nationwide data from the National Health Insurance Service (NHIS).

## Materials and methods

### Study population and design

This cohort study obtained data from the Korean National Health Insurance Service (K-NHIS) database. The Korean NHIS covers approximately 97% of Koreans, while the 3% of remaining Koreans who cannot afford national insurance are covered by the Medical Aid Program [20]. Therefore, the K-NHIS database represents the entire population of South Korea and contained national records of all covered inpatient and outpatient visits, diagnosis, procedures, and medical devices [21–23]. The NHIS routinely audits claims data, which have been widely used in population-based health research [22, 23]. The NHIS official review committee approved this study and permitted to access the data.

**Fig. 1:**
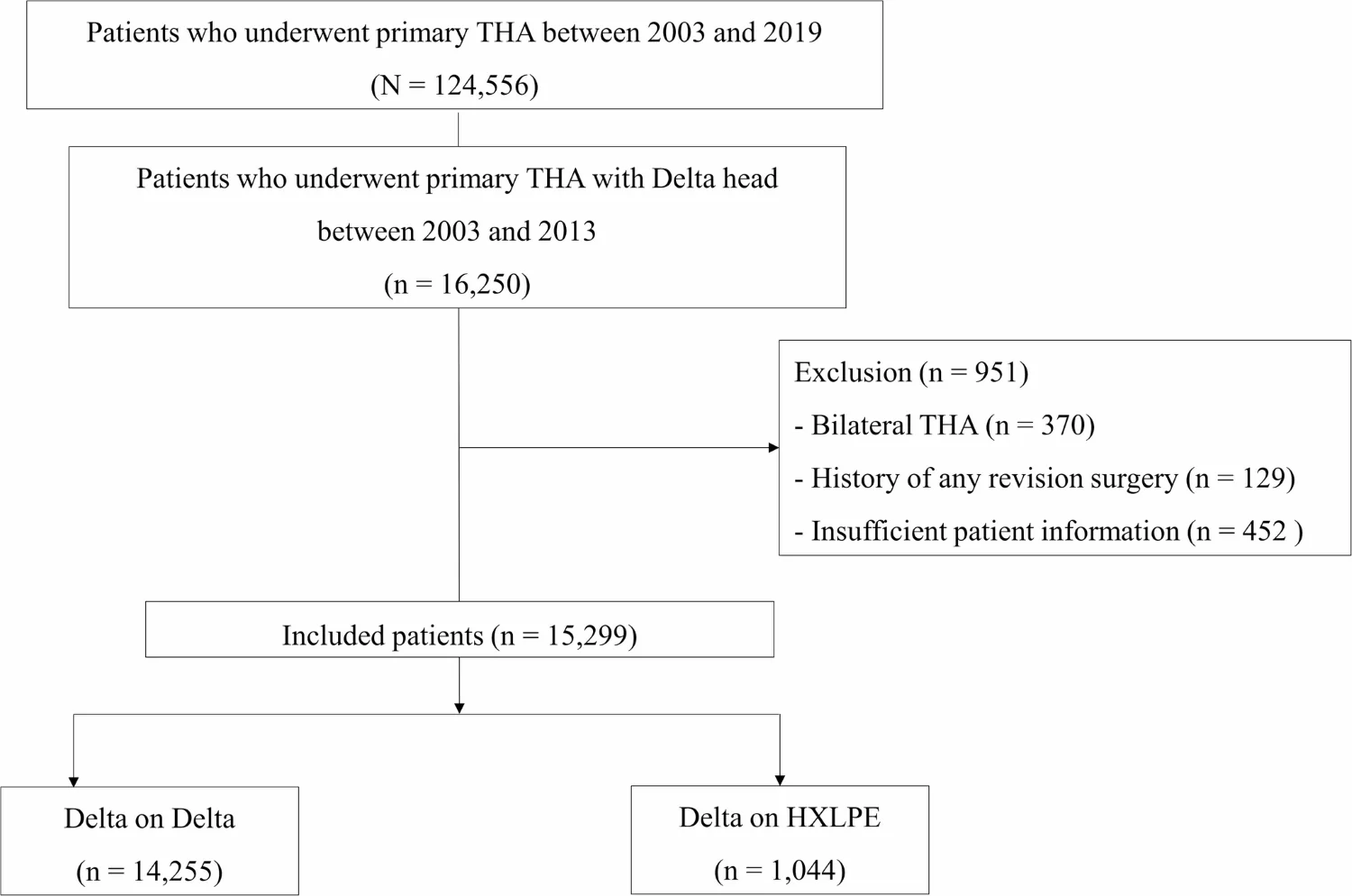
Flowchart of the study population. THA, total hip arthroplasty; DoD, delta-on-delta; DoHXLPE, delta-on-highly cross-linked polyethylene.

 For the THA registry, we included all adult patients older than 19 years who were admitted and underwent primary THA (procedure code: N0711, N2070) between January 1, 2003, and December 31, 2019 (*N* = 124,556). Among them, we selected patients who underwent primary THA using Delta ceramic head between January 1, 2003, and December 31, 2013 (*n* = 16,250). Those who had history of any revision surgery (*n* = 129) and bilateral THA (*n* = 370) were excluded. And patients with insufficient information (*n* = 452) were also excluded. Finally, a total of 15,299 THAs were included and analyzed in this registry study (Fig. 1).

### Type of bearings

To identify type of bearings, we reviewed all product codes of femoral head and liner that have been registered in Health Insurance Review and Assessment Service (HIRA) during study periods.

Delta ceramic was defined as zirconia-toughened alumina matrix composite ceramic. The HXLPE was defined as highly cross-linked polyethylene liner. According to the product codes of bearings, DoD and DoHXLPE group were categorized.

### Study outcomes

As clinical outcomes, dislocation was identified using a procedure code (N0761) and ICD-10 diagnosis codes (T840.20 and S730) [24, 25], whereas PJI (T845, T847, and T857) [26] and periprosthetic fracture (M966) [27] were identified using ICD-10 diagnosis codes. These outcomes were compared within 30 days, 90 days, and 1 year. The survivorships of THA with different type of liners were evaluated with 3 different end points: (1) stem revision, (2) cup revision, and (3) any revision. Stem revision was defined as the revision procedural code with the presence of product codes of femoral stems in the revision surgery. Cup revision was defined as the revision procedural code with the presence of product codes of acetabular cups. Any revision was defined as the presence of revision procedural codes regardless of product codes (N1711, N3710, N3720, N1715, N4710, N1725, N4720 and N1721). Death was treated as a censoring event. Vital status was obtained from death certification collected by Statistics Korea at the Ministry of Strategy and Finance of South Korea [22]. 

### Confounding factors

Age, and sex were obtained from the insurance eligibility database. Comorbidities within the past year prior to hospitalization were summarized using the Charlson index [28, 29]. We also included hypertension (ICD-10 code: I10- I13, I15) which are not included in the Charlson index. Reason of surgery was also defined using claim code at the admission. Hospitals were classified according to their capacity based on number of hospital beds and number of specialties as defined by the Korean Health Law [30]. Hospital is defined as a healthcare institution with more than 30 inpatient beds. General hospital is a hospital with more than 100 beds and more than 7 specialty departments including Internal Medicine, Surgery, Pediatrics, Obstetrics and Gynecology, Anesthesiology, Pathology, and Laboratory Medicine. Tertiary hospital is a general hospital with more than 20 specialty departments that serves as teaching hospital to medical students and nurses.

### Statistical analysis

Baseline differences between groups were evaluated using absolute standardized mean differences (SMDs). In line with commonly used conventions, values greater than 0.1 were considered to indicate meaningful imbalance. Categorical variables were assessed using chi-square or Fisher’s exact tests for statistical significance. Continuous data were analyzed with the student’s t-test where applicable.

For assessing outcomes, person-time was calculated from the surgery date until the occurrence of an outcome, death, or the end of the study period (until December 31, 2020), whichever occurred first. To evaluate cumulative incidence rate, we calculated within 30-, 90- and 1-years cumulative incidence of each outcome using Kaplan-Meier methods. Survival curves were generated by the Kaplan-Meier product-limit method and compared using log-rank tests. Adjusted hazard ratios (aHRs) and corresponding 95% confidence intervals (CIs) were calculated using Cox proportional hazards models. Proportionality of hazards was assessed through visual inspection of log-minus-log plots and Schoenfeld residuals. The models were adjusted for age, sex, level of hospital, hypertension and Charlson comorbidity index.

Since the main differences between two groups were observed for age and hospital type, mortality differed over long-term follow-up and could act as a competing event for revision-related outcomes. Thus, we performed competing risk analyses using the Fine and Gray method in addition to multivariable Cox regression adjusted for age, sex, year of surgery, hospital level, reason for surgery, hypertension, and Charlson comorbidity index. Age-stratified analyses of revision outcomes were additionally performed using 70 years as the cutoff. In addition, to address potential confounding, we performed 1:3 propensity score matching using a nearest-neighbor approach based on baseline demographic, clinical, and hospital-level variables. Balance between groups was reassessed using absolute standardized mean differences, with values greater than 0.1 considered to indicate meaningful imbalance.

All analyses were two sided and *P*-values < 0.05 were considered statistically significant. Statistical analyses were performed using SAS version 9.2 (SAS Institute, Inc, Cary, NC, USA) and R software, version 3.3.2 (Free Software Foundation, Inc., Boston, MA, USA).

This study was approved by the Institutional Review Board of the Samsung Medical Center (IRB number: SMC 2021-08-174), and the requirement for informed consent was waived as only de-identified data were used.

## Results

A total of 15,299 patients underwent THA with Delta ceramic head between 2003 and 2013.

**Fig. 2:**
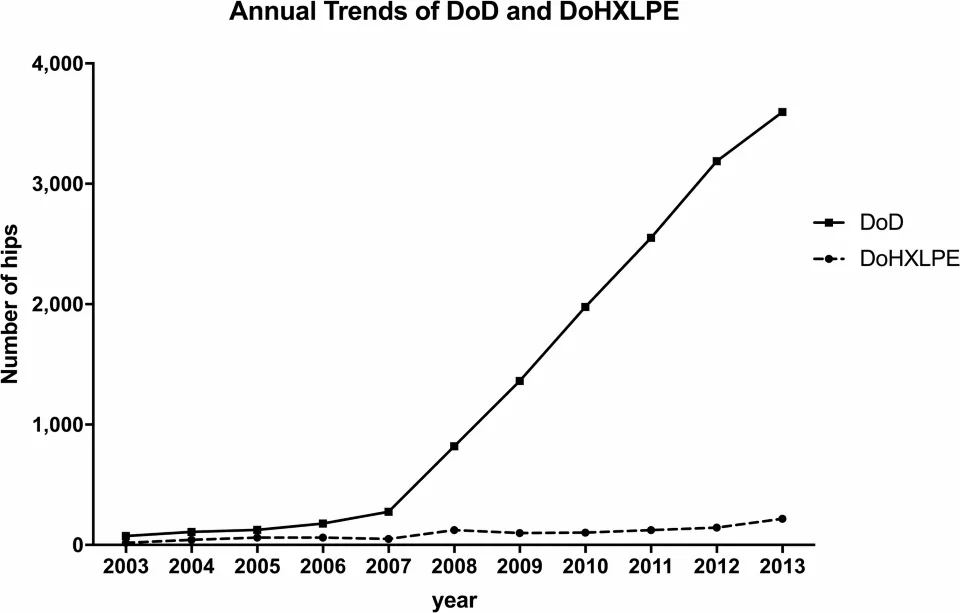
Annual trends in the use of DoD and DoHXLPE bearings in total hip arthroplasty. DoD, delta-on-delta; DoHXLPE, delta-on-highly cross-linkedpolyethylene.

 Delta liner was used in 14,255 patients (93.2%) and HXLPE in 1,044 patients (6.8%) respectively. A noticeable trend in liner utilization was observed over the study period, with an increase in Delta liner utilization since 2007 (Fig. 2).

Compared with the DoD group, patients receiving THA with the DoHXLPE bearing were older and more often female. Using an SMD threshold of 0.1, meaningful baseline imbalance was observed in several variables, including age, sex, hospital type, and Charlson comorbidity index. Osteonecrosis of femoral head was the most common cause of THA in both groups. (Table 1). In the propensity score–matched cohort, baseline imbalance was substantially improved compared with the original cohort, although some residual imbalance remained in several covariates (Supplementary Table 1).

**Table 1:** Baseline characteristics

	DoD (n = 14,255)	DoHXLPE (n = 1,044)	SMD
Age (years)	58.4 ± 13.7	64.3 ± 11.9	0.458
*Sex (%)*			−0.182
Male	7,573 (53.1)	460 (44.1)	
Female	6,682 (46.9)	584 (55.9)	
*Type of hospital (%)*			
Tertiary hospital	6,532 (45.8)	523 (50.1)	0.086
General hospital	4,258 (29.9)	162 (15.5)	−0.348
Hospital	3,303 (23.2)	358 (34.3)	0.248
Clinics	162 (1.1)	1 (0.1)	−0.133
Charlson Comorbidity Index	2.1 ± 1.9	2.3 ± 1.9	0.120
*Medical Comorbidity*			
Myocardial infarction (%)	187 (1.3)	17 (1.6)	0.026
Congestive heart failure (%)	665 (4.7)	79 (7.6)	0.121
Peripheral vascular disease (%)	2244 (15.7)	272 (26.1)	0.256
Cerebrovascular accident (%)	1352 (9.5)	151 (14.5)	0.154
COPD (%)	4132 (29.0)	379 (36.3)	0.157
Connective tissue disease (%)	2067 (14.5)	146 (14.0)	0.015
Peptic ulcer disease (%)	4463 (31.3)	430 (41.2)	0.207
Liver disease (%)	4473 (31.4)	281 (26.9)	0.098
Renal disease (%)	468 (3.3)	25 (2.4)	0.054
Hypertension, yes (%)	7406 (52.0)	588 (56.3)	0.088
*Reason of surgery (%)*			
Osteonecrosis of femoral head (%)	7541 (52.9)	522 (50.0)	0.058
Degenerative arthritis (%)	3349 (23.5)	343 (32.9)	0.209
Femoral neck fracture (%)	1387 (9.7)	50 (4.8)	−0.191
Secondary OA (%)	728 (5.1)	31 (3.0)	0.109
Hip dysplasia (%)	448 (3.1)	49 (4.7)	0.080
Inflammatory arthritis (%)	218 (1.5)	14 (1.3)	−0.086
Intertrochanteric fracture (%)	244 (1.7)	8 (0.8)	−0.082
Posttraumatic arthritis (%)	166 (1.2)	12 (1.1)	0.001
Septic hip sequelae (%)	107 (0.8)	13 (1.2)	−0.001
LCP sequelae (%)	48 (0.3)	0 (0.0)	−0.109
Acetabular fracture (%)	19 (0.1)	2 (0.2)	0.050
Follow-up (years)	8.7 ± 2.9	9.3 ± 3.7	0.180

*DoD* delta-on-delta, *DoHXLPE* delta-on-highly cross-linked polyethylene, *OA* osteoarthritis, *LCP* Legg-Calves-Perthes disease, *SMD* standardized mean difference

DoHXLPE group had a higher risk of PJI (aHR = 2.10, 95% CI = 1.29–3.41) compared to the DoD group within 1 year. (Table 2). However, there were no significant differences in risk of dislocation (aHR = 1.41, 95% CI = 0.97–2.05) and periprosthetic fracture (aHR = 0.90; 95% CI = 0.39, 2.07) within 1 year between DoD group and DoHXLPE group. Competing risk analyses accounting for mortality yielded similar subdistribution hazard estimates for these outcomes (Table 2). In the matched cohort, the DoHXLPE group remained at higher risk of dislocation (adjusted HR 1.68, 95% CI 1.08–2.63) and periprosthetic joint infection (adjusted HR 2.23, 95% CI 1.22–4.05), whereas the risk of periprosthetic fracture was not significantly different (adjusted HR 1.36, 95% CI 0.52–3.58) (Supplementary Table 2).

**Table 2:** Cumulative incidence rate and adjusted hazard ratio of complications within 30, 90 days and 1 year

Outcomes	Within 30 days			Within 90 days			Within 1 year		
	Event (Cumulative incidence rate)	Adjusted† HR	SubHRs (95% CI) †*	Event (Cumulative incidence rate)	Adjusted HR	SubHRs (95% CI) †*	Event (Cumulative incidence rate)	Adjusted HR	SubHRs (95% CI) †*
*Dislocation*									
DoD	98 (0.7)	*Reference*	**Reference**	222 (1.7)	*Reference*	**Reference**	315 (2.3)	*Reference*	**Reference**
DoHXLPE	9 (0.9)	1.18 (0.59, 2.36)	1.18 (0.59, 2.34)	22 (2.2)	1.32 (0.85, 2.07)	1.32 (0.85, 2.05)	31 (3.0)	1.41 (0.97, 2.05)	1.41 (0.97, 2.05)
*Periprosthetic joint infection*									
DoD	42 (0.3)	*Reference*	**Reference**	88 (0.6)	*Reference*	**Reference**	126 (0.9)	*Reference*	**Reference**
DoHXLPE	5 (0.5)	1.33 (0.52, 3.44)	1.33 (0.54, 3.32)	10 (1.0)	1.45 (0.74, 2.82)	1.45 (0.74, 2.82)	20 (1.9)	2.10 (1.29, 3.41)	2.10 (1.29, 3.43)
*Periprosthetic fracture*									
DoD	34 (0.2)	*Reference*	**Reference**	60 (0.4)	*Reference*	**Reference**	101 (0.7)	*Reference*	**Reference**
DoHXLPE	1 (0.0)	0.42 (0.06, 3.14)	0.42 (0.06, 3.12)	2 (0.2)	0.55 (0.13, 2.27)	0.55 (0.13, 2.26)	6 (0.6)	0.90 (0.39, 2.07)	0.90 (0.39, 2.06)

*DoD* delta-on-delta, *DoHXLPE* delta-on-highly cross-linked polyethylene, *HR* hazard ratio

*Death was considered as competing events

†Adjusted for age, sex, year of surgery, level of hospital, reason of surgery, hypertension and Charlson comorbidity index

At 10 years, the DoHXLPE group had a higher risk of any revision than the DoD group (aHR = 1.32; 95% CI, 1.03–1.38), whereas neither cup revision nor stem revision differed significantly between the groups. (Table 3). In age-stratified analyses, DoHXLPE was associated with a higher risk of any revision among patients aged 70 years or younger (adjusted HR 1.49, 95% CI 1.02–2.16). The point estimate was also above 1 among patients older than 70 years, although the confidence interval included unity (adjusted HR 1.38, 95% CI 0.84–2.26) (Supplementary Table 3). In the matched cohort, the point estimate for any revision remained higher in the DoHXLPE group (adjusted HR 1.52, 95% CI 0.82–2.84), although this difference did not reach statistical significance (Supplementary Table 4).

**Table 3:** 10-year cumulative incidence and adjusted hazard ratio of revision between DoD and DoHXLPE bearings

Outcomes	No of case (%)	Adjusted HR (95% CI)	SubHRs (95% CI) †*
*Cup revision*			
DoD	185 (1.4)	Reference	*Reference*
DoHXLPE	20 (1.9)	1.38 (0.86, 2.22)	1.40 (0.86, 2.27)
*Stem revision*			
DoD	161 (1.2)	Reference	*Reference*
DoHXLPE	16 (1.5)	1.37 (0.81, 2.32)	1.38 (0.82, 2.32)
*Any revision*			
DoD	468 (3.5)	Reference	*Reference*
DoHXLPE	51 (4.8)	1.32 (1.03, 1.38)	1.40 (1.03, 1.88)

†Adjusted for age, sex, year of surgery, level of hospital, reason of surgery, hypertension and Charlson comorbidity index

*Death was considered as competing events

DoD, delta-on-delta; DoHXLPE; delta-on-highly cross-linked polyethylene; No, number; HR, hazard ratio

**Fig. 3:**
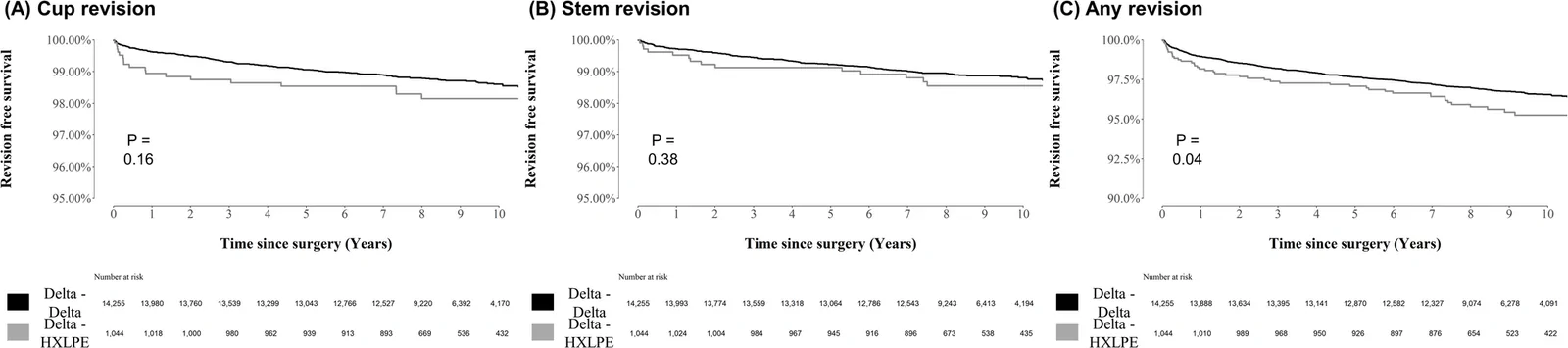
Kaplan–Meier survivorship curves according to bearing type for (a) cup revision, (b) stem revision, and (c) any revision. DoD, delta-on-delta; DoHXLPE, delta-on-highly cross-linked polyethylene.

 Ten-year Kaplan–Meier survivorship estimates for cup revision, stem revision, and any revision in both groups are presented in Table 4; Fig. 3.

**Table 4:** 10-year survivorship with each revision as endpoint.\

Type of liner	Cup revision	Stem revision	Any revision
DoD	98.6 (98.4, 98.8)	98.8 (98.6, 99.0)	96.5 (96.2, 96.9)
DoHXLPE	98.1 (97.3, 99.0)	98.5 (97.8, 99.3)	95.3 (93.9, 96.7)

*DoD* delta-on-delta, *DoHXLPE* delta-on-highly cross-linked polyethylene

## Discussion

To our knowledge, this is the first national registry–based cohort study to directly compare DoD with DoHXLPE bearings in an East Asian population. We found that the DoHXLPE group had a higher risk of PJI than the DoD group. The primary analysis suggested a higher risk of any revision in the DoHXLPE group; however, this association was not statistically significant after propensity score matching. Although the point estimate remained higher in the DoHXLPE group, the wide confidence interval in the matched cohort indicated insufficient evidence to conclude that the risk of any revision differed between the bearing groups.

The use of Delta liners increased markedly after their introduction in Korea in 2007. This increasing adoption may reflect advances in implant technology and surgeon preference, particularly because the two bearing types are similarly priced under the Korean reimbursement system. Delta liner was more frequently used in younger patients, patients with fewer comorbidities, and male patients. The marked imbalance in group size reflected the real-world bearing utilization pattern in Korea, as the cohort included all eligible THAs using a Delta ceramic head. Nevertheless, the smaller DoHXLPE cohort limited statistical precision, particularly for rare and long-term outcomes.

One notable finding was the higher observed risk of PJI in the DoHXLPE group. The biological basis for this difference remains uncertain. Previous registry-based studies have also reported associations between bearing material and the risk of infection-related revision [17, 18, 31]. However, our claims database lacks the clinical, microbiological, and operative information required to evaluate the underlying mechanisms. Therefore, this finding should be interpreted as an association rather than a causal effect of bearing material and may reflect residual or unmeasured confounding.

Another notable finding was the higher adjusted risk of dislocation in the DoHXLPE group in the propensity score–matched analysis. The reason for this association remains uncertain because dislocation after THA is multifactorial and may be influenced by femoral head size, surgical approach, component positioning, and surgeon-related factors [24, 25, 32, 33]. Notably, an Italian registry study found no significant difference in revision for dislocation between contemporary DoD and DoHXLPE bearings after adjustment for age, sex, and head size [32]. Because these technical variables were unavailable in the NHIS database, the observed association may reflect confounding by indication or other residual confounding rather than a direct effect of bearing material and should be interpreted cautiously.

Previous randomized and registry-based studies reported generally comparable outcomes between CoC and ceramic-on-HXLPE bearings but did not specifically compare contemporary DoD and DoHXLPE bearings in a nationwide cohort [18, 19, 34] (Table 5). In a study using data from the National Joint Registry of England and Wales, the reported hazard ratios relative to MoHXLPE indirectly suggested a higher 10-year risk of any revision for DoD than for DoHXLPE [34]. In our primary analysis, DoHXLPE was associated with a higher risk of any revision, whereas the risks of cup and stem revision were similar between the groups. However, the association with any revision was no longer statistically significant after propensity score matching. Furthermore, our “any revision” endpoint was an all-cause measure based on revision procedural codes and did not identify the specific indication for revision. Therefore, differences in endpoint definition and residual confounding should be considered when comparing our findings with those of previous registry studies.

**Table 5:** Risks of dislocation, infection, and overall revision of CoHXLPE bearings compared to CoC bearings in registry studies

Author	Nation/region	Study period	FU duration (years)	Number	Dislocation	Infection	Any revision
Madanat et al. [17]	Australia	1999–2013		CoC 57,839 CoHXLPE 24,269	NA	Reference 1.42(1.15, 1.75)	NA
Peters et al. [18]	Netherlands	2007–2016	9	CoC 17,625 CoHXLPE 70,175	3.4% 0.7%	0.3% 0.5%	4.1% (3.4–4.9) 4.0% (3.6–4.4)
Sharplin et al. [19]	New Zealand	1999–2015	10	CoC 11,235 CoHXLPE 14,382	0.5% 0.5%	0.4% 0.3%	0.61 (0.54–0.67)* 0.54 (0.48–0.61)*
Whitehouse et al. [31]	England and Wales	2003–2019	10	MoHXLPE 183,106 DoD 119,558 DoHXLPE 140,539	NA	NA	Reference 0.96(0.90–1.03) 0.79(0.73–0.85)
Present study	South Korea	2003–2013	10	DoD 14,255 DoHXLPE 1,044	Reference 1.41 (0.97, 2.05)	Reference 2.10 (1.29, 3.41)	Reference 1.32 (1.03, 1.38)

*CoC* ceramic-on-ceramic, *CoHXLPE* ceramic-on-highly cross-linked polyethylene, *MoHXLPE* metal-on-highly cross-linked polyethylene, *DoD* delta-on-delta, *DoHXLPE* delta-on-highly cross-linked polyethylene, *NA* not accessible

*per 100-implant year

Our study has several limitations. First, this was a non-randomized retrospective comparison and was therefore subject to selection bias. Although propensity score matching substantially improved baseline covariate balance, residual imbalance and unmeasured confounding remained. Therefore, the observed associations should not be interpreted as causal or as evidence of the superiority of either bearing. In addition, the smaller size of the DoHXLPE group and the limited number of outcome events reduced statistical precision, particularly in the propensity score–matched analyses. Second, the claims database lacked detailed clinical, operative, and radiographic information, including BMI, femoral head size, fixation method, surgical approach, operative time, implant positioning, and surgeon-related factors. These unavailable variables may have influenced bearing selection and postoperative outcomes, and their effects could not be evaluated. In addition, the study outcomes could not be independently adjudicated using medical records or imaging, and coding-related misclassification cannot be excluded. Third, generalizability may be limited because bearing prices are government-regulated and similar in Korea, unlike in other healthcare settings. Fourth, CoC-specific complications, including squeaking, ceramic fracture, and liner malseating, could not be captured in the claims data. Fifth, “any revision” was an all-cause endpoint encompassing revisions performed for heterogeneous indications. Although cup and stem revisions were analyzed separately, the specific indications for revision could not be determined from the available claims data; therefore, findings for this endpoint should be interpreted cautiously. Future prospective studies incorporating detailed clinical and procedural information are warranted.

Despite these limitations, our study provides additional nationwide evidence regarding the comparative outcomes of DoD and DoHXLPE bearings. In this nationwide observational cohort, DoHXLPE was associated with a higher observed risk of PJI compared with DoD. Although the primary analysis also suggested a higher risk of any revision, this finding was not statistically significant after propensity score matching. This study does not establish causality or demonstrate the superiority of either bearing, and further studies incorporating more detailed clinical and procedural variables are warranted.

## Supplementary Information

Below is the link to the electronic supplementary material.

Supplementary Material 1

Supplementary Material 2

Supplementary Material 3

Supplementary Material 4

## Data Availability

No datasets were generated or analysed during the current study.
