# Supplementary material for: Comparative outcomes of delta-on-delta and delta-on-highly cross-linked polyethylene bearings in total hip arthroplasty: a nationwide cohort study
Source: Arch Orthop Trauma Surg. 2026 Sep 18;146(1):338. doi: 10.1007/s00402-026-06502-1 (PMC13588854; doi:10.1007/s00402-026-06502-1)
Supplement: Supplementary file 1 [file 402_2026_6502_MOESM1_ESM.docx]

**Supplementary Table 1. Baseline Characteristics After PS matching (N = 4,167)**

|  | DoD  (N = 3,123) | DoHXLPE  (N = 1,044) | SMD |
| --- | --- | --- | --- |
| Age (years) | 63.9 ± 12.5 | 64.3 ± 11.9 | 0.033 |
| Sex (%) |  |  | 0.000 |
| Male | 1,376 (44.1) | 460 (44.1) |  |
| Female | 1,747 (55.9) | 584 (55.9) |  |
| Type of hospital (%) |  |  |  |
| Tertiary hospital | 1,695 (54.3) | 523 (50.1) | -0.084 |
| General hospital | 445 (14.2) | 162 (15.5) | 0.036 |
| Hospital | 980 (31.4) | 358 (34.3) | 0.062 |
| Clinics | 3 (0.1) | 1 (0.1) | 0.000 |
| Charlson Comorbidity Index | 2.2 ± 2.0 | 2.3 ± 1.9 | 0.051 |
| Medical Comorbidity |  |  |  |
| Myocardial infarction (%) | 47 (1.5) | 17 (1.6) | 0.010 |
| Congestive heart failure (%) | 189 (6.1) | 79 (7.6) | 0.060 |
| Peripheral vascular disease (%) | 582 (18.6) | 272 (26.1) | 0.179 |
| Cerebrovascular accident (%) | 335 (10.7) | 151 (14.5) | 0.113 |
| COPD (%) | 1,006 (32.2) | 379 (36.3) | 0.086 |
| Connective tissue disease (%) | 462 (14.8) | 146 (14.0) | -0.023 |
| Peptic ulcer disease (%) | 1,112 (35.6) | 430 (41.2) | 0.115 |
| Liver disease (%) | 896 (28.7) | 281 (26.9) | -0.040 |
| Renal disease (%) | 82 (2.6) | 25 (2.4) | 0.015 |
| Hypertension, yes (%) | 1,717 (55.0) | 588 (56.3) | 0.027 |
| Reason of surgery (%) |  |  |  |
| Osteonecrosis of femoral head (%) | 1,653 (52.9) | 522 (50.0) | -0.059 |
| Degenerative arthritis (%) | 984 (31.5) | 343 (32.9) | 0.029 |
| Femoral neck fracture (%) | 136 (4.4) | 50 (4.8) | 0.021 |
| Secondary OA (others) (%) | 149 (4.8) | 31 (3.0) | -0.094 |
| Hip Dysplasia (%) | 91 (2.9) | 49 (4.7) | 0.093 |
| Inflammatory arthritis (%) | 37 (1.3) | 14 (1.3) | 0.014 |
| Intertrochanteric fracture (%) | 16 (0.5) | 8 (0.8) | 0.032 |
| Posttraumatic arthritis (%) | 29 (0.9) | 12 (1.1) | 0.022 |
| Septic hip sequelae (%) | 19 (0.6) | 13 (1.2) | 0.066 |
| LCP sequelae (%) | 7 (0.2) | 0 (0.0) | -0.067 |
| Acetabular fracture (%) | 2 (0.1) | 2 (0.2) | 0.036 |
| Follow-up (years) | 8.7 ± 2.9 | 9.3 ± 3.7 | -0.180 |
