# Supplementary material for: Comparative outcomes of delta-on-delta and delta-on-highly cross-linked polyethylene bearings in total hip arthroplasty: a nationwide cohort study
Source: Arch Orthop Trauma Surg. 2026 Sep 18;146(1):338. doi: 10.1007/s00402-026-06502-1 (PMC13588854; doi:10.1007/s00402-026-06502-1)
Supplement: Supplementary file 2 [file 402_2026_6502_MOESM2_ESM.docx]

**Supplementary Table 2. Cumulative incidence rate and hazard ratio of clinical outcomes within 1 year after PS matching**

| **Outcomes** | **Within 1 year** | |
| --- | --- | --- |
|  | **Event**  **(Cumulative**  **incidence rate)** | **Adjusted**  **HR** |
| **Dislocation** |  |  |
| DoD | 53 (3.5) | *Reference* |
| DoHXLPE | 31 (3.1) | **1.68 (1.08, 2.63)** |
| **Periprosthetic joint infection** |  |  |
| DoD | 25 (1.2) | *Reference* |
| DoHXLPE | 19 (2.0) | **2.23 (1.22, 4.05)** |
| **Periprosthetic fracture** |  |  |
| DoD | 13 (0.7) | *Reference* |
| DoHXLPE | 6 (0.6) | 1.36 (0.52, 3.58) |

†Adjusted for age, sex, year of surgery, level of hospital, reason of surgery, hypertension and Charlson comorbidity index

DoD, Delta-on-delta; DoHXLPE, Delta-on-highly crosslinked polyethylene
