# Supplementary material for: Comparative outcomes of delta-on-delta and delta-on-highly cross-linked polyethylene bearings in total hip arthroplasty: a nationwide cohort study
Source: Arch Orthop Trauma Surg. 2026 Sep 18;146(1):338. doi: 10.1007/s00402-026-06502-1 (PMC13588854; doi:10.1007/s00402-026-06502-1)
Supplement: Supplementary file 3 [file 402_2026_6502_MOESM3_ESM.docx]

**Supplementary Table 3. Age-stratified adjusted hazard ratios for 10-year revision outcomes by bearing type**

| **Outcomes** | **Adjusted**† **HR (95% CI)** | |
| --- | --- | --- |
|  | **Age ≤ 70** | **Age > 70** |
| **Cup revision (cup included)** |  |  |
| Delta – Delta | *Reference* | *Reference* |
| Delta – HXLPE | 1.17 (0.61, 2.23) | 1.78 (0.86, 3.67) |
| **Stem revision (stem included)** |  |  |
| Delta – Delta | *Reference* | *Reference* |
| Delta – HXLPE | 1.56 (0.83, 2.92) | 1.10 (0.42, 2.85) |
| **Any revision** |  |  |
| Delta – Delta | *Reference* | *Reference* |
| Delta – HXLPE | 1.49 (1.02, 2.16) | 1.38 (0.84, 2.26) |

†Adjusted for age, sex, year of surgery, level of hospital, reason of surgery, hypertension and Charlson comorbidity index

DoD, delta-on-delta; DoHXLPE; delta-on-highly cross-linked polyethylene; No, number; HR, hazard ratio
