# Supplementary material for: Comparative outcomes of delta-on-delta and delta-on-highly cross-linked polyethylene bearings in total hip arthroplasty: a nationwide cohort study
Source: Arch Orthop Trauma Surg. 2026 Sep 18;146(1):338. doi: 10.1007/s00402-026-06502-1 (PMC13588854; doi:10.1007/s00402-026-06502-1)
Supplement: Supplementary file 4 [file 402_2026_6502_MOESM4_ESM.docx]

**Supplementary Table 4. 10-year cumulative incidence and adjusted hazard ratio of any revision of DoD and DoHXLPE After PS matching**

| **Outcomes** | **No of case (%)** | **Adjusted**† **HR (95% CI)** |
| --- | --- | --- |
| **Any revision** |  |  |
| DoD | 36 (1.4) | *Reference* |
| DoHXLPE | 20 (1.9) | 1.52 (0.82, 2.84) |

†Adjusted for age, sex, year of surgery, level of hospital, reason of surgery, hypertension and Charlson comorbidity index

DoD, delta-on-delta; DoHXLPE, Delta-on-highly crosslinked polyethylene
